# Bioperformance Studies of Biphasic Calcium Phosphate Scaffolds Extracted from Fish Bones Impregnated with Free Curcumin and Complexed with β-Cyclodextrin in Bone Regeneration

**DOI:** 10.3390/biom12030383

**Published:** 2022-02-28

**Authors:** Cecilia V. R. Truite, Jessica N. G. Noronha, Gabriela C. Prado, Leonardo N. Santos, Raquel S. Palácios, Adriane do Nascimento, Eduardo A. Volnistem, Thamara T. da Silva Crozatti, Carolina P. Francisco, Francielle Sato, Wilson R. Weinand, Luzmarina Hernandes, Graciette Matioli

**Affiliations:** 1 Department of Pharmacy, State University of Maringá (UEM), 5790 Colombo Avenue, Maringá 87020-900, PR, Brazil; 2 Department of Morphological Sciences, State University of Maringá (UEM), 5790 Colombo Avenue, Maringá 87020-900, PR, Brazil; jessicangnoronha@gmail.com (J.N.G.N.); gacprado@gmail.com (G.C.P.); ra106968@uem.br (L.N.S.); luzhernandes@gmail.com (L.H.); 3 Department of Physics, State University of Maringá (UEM), 5790 Colombo Avenue, Maringá 87020-900, PR, Brazil; pg53643@uem.br (R.S.P.); adriane.fisica@gmail.com (A.d.N.); e.volnistem@gmail.com (E.A.V.); fransatou@gmail.com (F.S.); wilson@dfi.uem.br (W.R.W.); 4 Department of Food Engineering, State University of Maringá (UEM), 5790 Colombo Avenue, Maringá 87020-900, PR, Brazil; thamarathaiane01@hotmail.com; 5 Department of Chemical Engineering, State University of Maringá (UEM), 5790 Colombo Avenue, Maringá 87020-900, PR, Brazil; carol_pfrancisco@hotmail.com

**Keywords:** biphasic calcium phosphate, biomaterials, bone regeneration, curcumin, β-cyclodextrin, materials engineering

## Abstract

Fish bones are a natural calcium phosphate (CaP) sources used in biomaterials production for bone regeneration. CaP scaffolds can be enriched with other substances with biological activity to improve bone repair. This study aimed to evaluate the physicochemical properties and bone regeneration potential of biphasic calcium phosphate (BCP) scaffolds impregnated with free curcumin (BCP-CL) or complexed with β-cyclodextrin (BCP-CD) compared to BCP scaffolds. Rietveld’s refinement showed that BCP is composed of 57.2% of HAp and 42.8% of β-TCP and the molar ratio of Ca/P corresponds to 1.59. The scaffolds presented porosity (macro and microporosity) of 57.21%. Apatite formation occurred on the BCP, BCP-CL, and BCP-CD surface, in vitro, in SBF. Micro-Raman technique showed a reduction in the dissolution rate of β-TCP in the curcumin-impregnated scaffolds over time, and in vivo studies on critical-size defects, in rat calvaria, had no additional regenerative effect of BCP-CL and BCP-CD scaffolds, compared to BCP scaffolds. Despite this, the study showed that curcumin impregnation in BCP scaffolds prolongs the release of the β-TCP phase, the BCP- phase with the higher osteoinductive potential, representing an advantage in tissue engineering.

## 1. Introduction

Biomaterials are used to restore, repair or replace injured tissues, promoting cell migration and adhesion, tissue growth and nutrient diffusion [1,2,3]. Their regenerative ability is associated with its physicochemical properties, such as crystallinity, solubility, phase composition, surface chemistry, ionic charge, roughness and porosity [4,5].

The most commonly used biomaterials for bone regeneration are the calcium phosphate-based ones. Hydroxyapatite (HAp) or biphasic calcium phosphate (BCP) are alternatives to autologous bone grafting (gold standard), and are widely used to promote bone regeneration due to their osteoinductive properties [6,7]. BCP, formed by mixing HAp with β-TCP (β-tricalcium phosphate), has a greater osteoinductive potential than HAp or β-TCP alone, causing mesenchymal cells to differentiate into osteoblasts with different properties, depending on the HAp/TCP ratio [8,9]. Studies show that the use of BCP has a more active biological action than pure HAp, as it presents characteristics closer to the HAp found in bones, leading to faster bone formation compared to HAp and β-TCP separately [10,11,12]. In vitro, BCP acts in a balanced process between resorption and bone formation, releasing calcium and phosphate ions into the microenvironment and precipitating a biological apatite on the ceramic surface, which is used to build the new bone [5,13,14].

Fluorapatite (FAP), wollastonite, diopside and tricalcium phosphate can be applied in bone regeneration [15,16]. Calcium phosphates (CaPs) can be synthesized [17] or obtained from natural sources. The Nile tilapia is considered to be the most widely produced freshwater fish species in the world. However, only 50 to 60% of the total catch is available for commercialization, while carcasses, dead or damaged fish are discarded, generating a large amount of solid waste thrown into the environment, which causes pollution and risks to public health [18]. The residues can be used for the production of calcium phosphate-based bioceramics, with excellent bioperformance for bone regeneration [19,20,21,22,23].

CaPs can be enriched with other types of substances with osteogenic properties, such as Bone Morphogenetic Protein-2 (BMP-2), platelet-rich plasma (PRP), growth factors (PDGF (Platelet-derived growth factor) and TGF-β1 (Transforming Growth Factor-β1)) [24,25] and other bioactive substances [26]. Studies show that some herbal medicines, such as curcumin, alone or associated with calcium phosphate ceramics can act as a bone regeneration stimulating agent, with beneficial effects in bone disorders and inflammatory diseases, including osteolysis, periodontitis, rheumatoid arthritis and osteoporosis [27,28].

Curcumin, found in the rhizomes of *Curcuma longa*, is a hydrophobic yellow-orange polyphenol that has been used for centuries as a spice and in pharmaceutical preparations [29]. It shows anti-inflammatory, antioxidant and antimicrobial functions. Besides this, it is being a potent inhibitor of nuclear factor-kβ (NF-kβ) and its ligand RANKL, thus stimulating the differentiation and mineralization of primary bone marrow cells and pre-osteoblast. Therefore, it is interesting to study the curcumin role in bone regeneration [30]. Son et al., [31] demonstrated that the use of curcumin, similarly to BMP-2 and growth factors, has the ability to induce osteoblast differentiation and increase the expression of osteocalcin, a marker of bone formation [31], in addition to preventing the deterioration of bone structure, resulting in beneficial changes in bone regeneration and growth [32,33].

However, the use of curcumin is limited by its low water solubility and rapid photodegradation. In order to improve its bioavailability [34], it can be complexed with β-cyclodextrins (β-CDs). β-CDs are cyclic oligosaccharides, with a hydrophobic cavity that hosts in its interior fat-soluble molecules. Therefore, a controlled release and increased absorption, the pharmacological properties are maintained and the biological effects maximized [35].

In this study, the physicochemical properties of BCP ceramics obtained from bones of the Nile tilapia fish (*Oreochromis niloticus*), in the form of powder and scaffolds were evaluated. Bone regeneration potential of BCP scaffolds impregnated with free curcumin (BCP-CL) or complexed with β-cyclodextrin (BCP-CD) compared to BCP scaffolds was investigated in in vitro studies in SBF (Simulated Body Fluid) and in vivo in critical defects in rat calvaria.

## 2. Materials and Methods

### 2.1. Obtaining Biphasic Calcium Phosphate (BCP)

The raw material used in the production of BCP was obtained from bones of the Nile tilapia fish (*Oreochromis niloticus*), aged between 90 and 100 d, from the Fish Culture Center of the Animal Science Department of the State University of Maringá (Department of Zootechnics—DZO-UEM). The fishes were raised in net tanks at the Rio do Corvo Station on the Diamante do Norte Regional Campus—UEM. The processing of the material was carried out in the Metallic Materials and Biomaterials Laboratory of the Physics Department of the State University of Maringá (Department of Physics—DFI-UEM), via calcination at 900 °C for 8 h followed by grinding in a Retsch PM 100 high energy mill (Haan, Germany) at 300 r.p.m. for 8 h, using a grinding jar and zirconia balls, and ball to powder mass ratio of 6/1, according to the procedures contained in PI0506242-0 [36].

### 2.2. Preparation of BCP Scaffolds

The BCP scaffolds were produced by powder metallurgy techniques using lactose as the space holder method [37]. The BCP powder particles (HAp+βTCP) were mixed with the spacer element in a 1:1 ratio (in vol%). The mixture was homogenized gently via mechanical vibration for 30 min and subsequently were uniaxially conformed using a rigid single-acting matrix and a PHP 30 TONs Metal PEM press (Maringá/Br), in a circular form with 8.1 mm in diameter and 2.5 mm in thickness. The samples were sintered at 1100 °C for 2 h in air atmosphere in a tube furnace. After sintering, the samples were sanded with 400 grit sandpaper until a 1 mm thickness was reached, washed in an ultrasonic bath for 10 min in acetone, alcohol and deionized water to eliminate sintering and sanding residues. The scaffolds were dried in a muffle furnace at 80 °C for 24 h and then kept in the desiccator [38].

### 2.3. Characterization of BCP Powder (HAp+β-TCP)

For the physicochemical characterization of the BCP powder, X-ray diffraction (XRD), Fourier transform infrared reflectance (FTIR) and micro-Raman analysis were performed. Some samples of the sintered scaffolds were broken and milled in agate mortar and then in a high-energy mill in air atmosphere for 1 h at 300 r.p.m., using a grinding jar and zirconia balls in a ball to powder mass ratio of 6:1.

### 2.4. Analysis of Morphology and Particle Size of BCP

For the analysis, the powder particles were subjected to an ultrasonic bath in acetone for 5 min to minimize particle aggregation effects. Particle size distribution analysis (%) was obtained on a Dynamic Light Scattering-DLS equipment (Malvern Instruments Ltd., London, UK) of the COMCAP-UEM (Research Support Center Complex, Maringá, Brazil).

The BCP powder morphology was evaluated by scanning electron microscopy (SEM) on a FEI Quanta 250 machine (ThermoFisher Scientific, Waltham, MA, USA), manufactured by Oxford Instruments, Oxon, UK. The samples were coated with a conductive gold film by sputtering in a Shimadzu IC-50 Ion Coater metallizer (Shimadzu Corporation, Kyoto, Japan) of the COMCAP-UEM.

### 2.5. X-ray Diffraction Analysis and Rietveld Refinement

A Shimadzu XRD 7000 X-ray diffractometer (Shimadzu Corporation, Kyoto, Japan) with Cu Kα irradiation source (λ = 0.15418 nm) was used to determine the phase composition. The data was collected at 40 kV, 30 mA between 10 and 80°, a scan rate of 0.5°/min. and angular increment of 0.02° of the COMCAP-UEM. The phases were identified by comparison with the JCPDS (Joint Committee Series of Powder Diffraction Standards) and ICSD (Database of Inorganic Crystal Structure) standards [39,40]. The Rietveld method was used to quantify the crystalline phases present in BCP using the FullProff program (Version 4.30—2008) [41,42].

### 2.6. BCP Powder Analysis by Infrared Spectroscopy (FTIR)

FTIR spectroscopy was used to investigate the compounds and functional groups of the precursor powder, using a Bruker Model Vertex 70v spectrometer (Bruker Optik GmbH, Ettelingen, Germany). For FTIR analysis, 2 mg of each sample was weighed and diluted in 198 mg of KBr to form the tablets. Each spectrum had an average of 128 scans, ranging from 400 to 4000 cm^−1^ with a resolution of 4 cm^−1^ of the COMCAP-UEM.

### 2.7. BCP Powder Analysis by Micro-Raman Spectroscopy

Micro-Raman spectroscopy measurements were performed on a Confocal Raman spectrometer, Bruker, Model Senterra (Bruker Optik GmbH, Ettelingen, Germany) at an optical magnification of 20×, using an excitation laser with a wavelength of 532 nm, power of 20 mW and 20 scans, acquired for 3 s in each exposure of the COMCAP-UEM.

### 2.8. Analysis of Scaffolds Porosity

The porosity of the scaffolds was determined by the liquid displacement method of Archimedes Principle, NORM ASTM C373-88 [43], using deionized water, which penetrated the pores without causing any dimensional changes in the sample. The scaffolds were dried for 24 h in an oven at 100 °C. After the samples were cold and dry, the measurements of the sintered dry mass (*m_s_*) were made. Next, the samples were placed in a chamber, in which vacuum was established between 10^−1^ and 10^−2^ torr for 2 h to force the liquid into the pores. After this period, the chamber was flooded and there was a 30 min wait at room temperature to measure the impregnated mass (*m_i_*) and the impregnated mass under thrust (*m_e_*), using a Shimadzu scale (AUW220D, Japan) and Shimadzu Specific Gravity Measurement Kit accessory (DFI-UEM). The porosity values correspond to the average of the values measured in six (06) samples. The open porosity ((γ (%)) was calculated by the Equation (1) where: γ (%) is open porosity; (*m_s_*) the mass of the sintered and dried sample; (*m_i_*) the mass of the sample impregnated with the liquid, and (*m_e_*) the mass of the sample impregnated under liquid thrust:

(1) $$\gamma \left(\%\right)=\left(\frac{m_{i}-m_{s}}{m_{i}-m_{e}}\right)\times 100$$

### 2.9. Analysis of BCP Scaffolds by Scanning Electron Microscopy (SEM)

The pores of the scaffolds were observed and evaluated by scanning electron microscopy (SEM) on an FEI Quanta 250 (ThermoFisher Scientific, Waltham, MA, USA), manufactured by Oxford Instruments, Oxon, UK. The samples were coated with a conductive gold film by sputtering in a Shimadzu IC-50 Ion Coater metallizer (Shimadzu Corporation, Kyoto, Japan).

### 2.10. Curcumin Complexation with β-Cyclodextrin (β-CD) by Coprecipitation Method and Incorporation in BCP Scaffolds

Curcumin and β-CD were purchased from Sigma (St. Louis, MO, USA). For the complexation process of curcumin and β-CD, a 0.06M solution of β-CD was placed in a 500 mL flat bottom flask, attached to the condenser, where it was used water at 4 °C for cooling. The magnetic stirrer was set for heating to 70 °C. Curcumin was dissolved in 60 °GL ethanol and added to the flask under dripping. The ethanol was completely removed using a rotary evaporator at 70 °C. Subsequently, the solution was placed in a reactor and stirred for 8 h at 25 °C and kept in a refrigerator for 12 h at a temperature of 2 to 4 °C. After filtering with white quantitative filter paper (porosity of 4 to 7 µm), the material was dried in an oven at 50–55 °C for at least 6h. The process was performed in the dark because curcumin degrades easily in light [35].

In order to obtain the free curcumin solution, 1mg/mL of curcumin was added in 70% alcohol. To obtain the solution of curcumin complexed with β-CD, 7.16 mg/mL of the complex was required to ensure that curcumin concentration corresponded to 1 mg/mL, equivalent to the 2:1 ratio of cyclodextrin:curcumin.

The scaffolds were soaked in 50 µL of the solutions of curcumin in free form and curcumin complexed with β-CD in a dark environment, dried and stored at room temperature, protected from light.

### 2.11. In Vitro Bioactivities Study of Scaffolds with Curcumin and Curcumin-β-CD

For the in vitro studies in simulated body fluid (SBF), the sintered scaffolds (control BCP); (BCP soaked in curcumin); (BCP soaked in curcumin-CD) were immersed in 30 mL of an acellular SBF with ionic concentrations: (Na^+^: 142.0; K^+^: 5.0; Ca^2+^: 2.5; Mg^2+^: 1.5; Cl^−:^ 147.8; HCO_3_^2−^: 4.2; HPO_4_^2−^: 1.0; SO_4_^2−^: 0.5 mM), close to those of human blood plasma. The reagents KCl, K_2_HPO_4_·3H_2_O, MgCl_2_·6H_2_O, CaCl_2_, Na_2_SO_4_ were pipetted into a polystyrene flask with ultrapure water at a (controlled) temperature of 36.5 °C and buffered with tris(hydroxymethyl)aminomethane and HCl to a pH of 7.4. The scaffolds were immersed (vertically) in 30 mL of SBF in falcon tubes and placed in a water bath at 36.5 °C for 28 d. After this immersion period, the samples were removed from the SBF, gently washed with ultrapure water and dried at 40 °C. The SBF solution was prepared according to the protocol suggested in the reference [44].

The morphology of the scaffolds after immersion in SBF was evaluated by scanning electron microscopy (SEM) under the same conditions as mentioned in 2.9.

An analysis of the sample composition was performed using an energy dispersive X-ray detector (EDS), which allows a qualitative and semi-quantitative analysis of the sample composition, enabling, besides the identification of the chemical elements present, the proportion between them [45].

### 2.12. In Vivo Bone Regeneration Study Evaluating Scaffolds Impregnated with Curcumin in Free Form and Complexed with Β-CD

All procedures involving the use of animals were approved by the Ethics Committee on Animal Experimentation of the State University of Maringá, filed under CEUA No. 3379090218.

Sixty adults male Wistar rats weighing between 200 and 250g from the Central Animal House of the State University of Maringá were used. During the experimental period, the animals received chow (Nuvilab^®^/Nuvital^®^, Sogorb^®^, São Paulo, Brazil) and water ad libitum.

After intramuscular anesthesia with 2% xylazine hydrochloride (1 mL/kg) and 10% ketamine (1 mL/kg) 1:1, trichotomy and antisepsis of the head region, an incision was made in the skin, up to the periosteum, from the base of one ear to the other, transversely across the callus. For complete bone exposure, the tissues were detached using a molt spatula.

Using an 8 mm diameter trephine drill (Neodent^®^, Curitiba, Brazil) on a straight piece (Kavo^®^, Joinville, Brazil), attached to a surgical motor (Branemark System^®^, Zurich, Switzerland) rotating at 1500 rpm and abundant irrigation with sterile saline solution, a bone defect of 0.8 mm deep and 8 mm in diameter was made in the calvaria.

The scaffolds were implanted in the defects and the flap was sutured with single stitches using Mononylon 4-0 thread (Ethicon^®^ Johnson, Bridgewater, NJ, USA). The area received topical application of an alcoholic solution of iodinated polyvinylpyrrolidone as a local antiseptic measure.

The animals were divided into three groups according to the chemical composition of the scaffolds implanted in the bone defect: (1) BCP Group, with pure BCP scaffolds (control); (2) BCP-CL group, with scaffolds impregnated with free curcumin, and (3) BCP-CD with scaffolds impregnated with curcumin impregnated with β-CD.

Euthanasia was performed 15, 30, 45 and 60 d after implantation, with injection of thiopental solution in overdose 120 mg/kg, and samples containing the scaffolds were analyzed.

#### 2.12.1. Micro-Raman Spectroscopy Analysis of the Scaffolds Implanted in the Calvaria of the Animals

The scaffold samples implanted in the calvaria of the animals (*n* = 1/time/group) were removed, cleaned with ultrapure water, dried for 24 h at room temperature, macerated and homogenized in agate mortar for micro-Raman spectroscopy analyses. Measurements were made in three different points of the samples in the Raman spectrum region between 1000 and 900 cm^−1^, according to the equipment and methodology described in item 2.7.

#### 2.12.2. Histology of the Scaffolds

For this study, the minimum number of three animals/time/group were used. In 60-d group: BCP, *n* = 3 animals; BCP-CL and BCP-CD, *n* = 5 animals. In 45-d group: BCP, *n* = 3 animals; BCP-CL and BCP-CD, *n* = 4 animals. In 30 and 15-d groups: BCP, BCP-CL and BCP-CD, *n* = 3 animals). Calvaria samples were collected, fixated in 4% paraformaldehyde and decalcified in EDTA. Then, the samples were divided in half and processed for paraffin embedding. Serial sections of 7–8 µm were made and stained with H&E to morphological study. For each sample, five slides containing four histological sections each were analyzed by two different observers.

## 3. Results

### 3.1. Analysis of the Morphology and Particle Size of BCP

**Figure 1:**
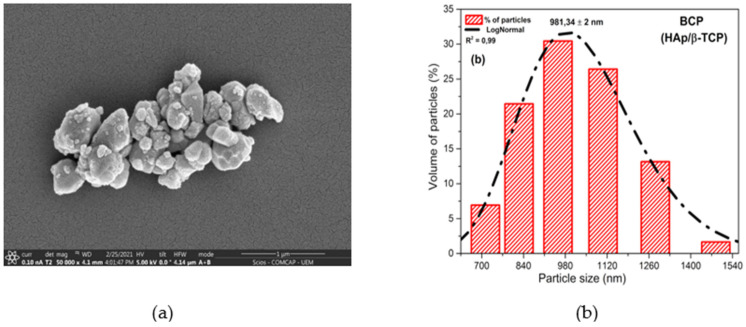
Morphology and particle size of BCP powder obtained by calcination fish bones at 900 °C for 8 h and milled in air atmosphere in a power mill for 4h at 300 r.p.m.: (**a**) SEM showing particle shape and size; (**b**) histogram relating frequency and particle size. R^2^ = Adjusted R-Square; BCP = biphasic calcium phosphate; HAp = hydroxyapatite; β-TCP = β-tricalcium phosphate.

Scanning electron microscopy (SEM) (Figure 1a) shows the powder morphology of the BCP after calcination at 900 °C. The image reveals weakly aggregated particles of different sizes and smaller than 1100 nm. The histogram presents in Figure 1b shows the particle size distribution between 712.4 and 1483.9 nm, with a center at 981.34 nm and the relation between frequency and particle size. Powder properties influence the microstructure and the physical and mechanical properties of sintered materials, with control of particle size and particle size distribution being important parameters in the processing of materials [46,47].

### 3.2. X-ray Diffraction Analysis and Rietveld Refinement

**Figure 2:**
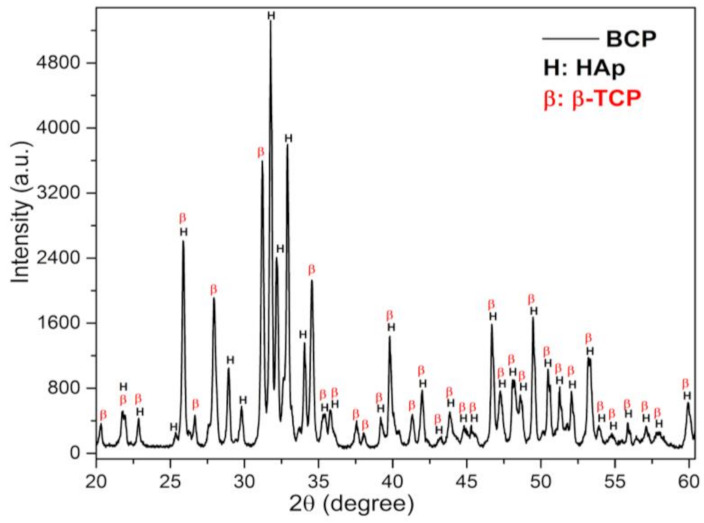
DRX pattern of BCP powder obtained by calcining fish bones at 900 °C for 8 h and milling in a high-energy mill for 4 h at 600 r.p.m. The peaks related to the phases, HAp and β-TCP, are indicated by the letters (H) and (B), respectively. BCP = biphasic calcium phosphate; HAp = Hydroxyapatite; β-TCP = β-tricalcium phosphate.

The X-ray diffraction (DRX) pattern of BCP powder after calcination and milling is shown in Figure 2. Characteristic peaks of the HAp (H) and β-TCP (β) phases are observed and have been indexed to the Joint Committee on Powder Diffraction Standards, jcpds 09-0432 and 09-0169, respectively [39,40]. No other minority phases were detected within the detection limits of the X-ray technique (~2%).

**Figure 3:**
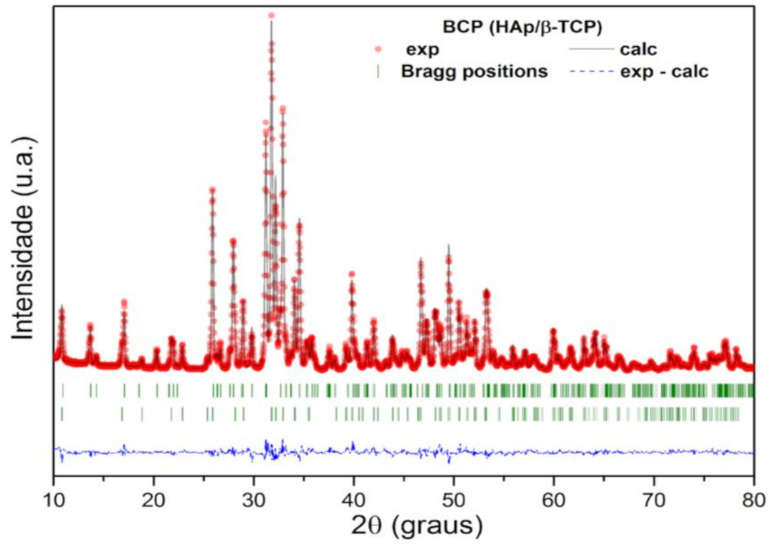
Rietveld refinement results for the calcined and milled BCP powder. The red circles are the experimental XRD data and the black solid line is the refinement result (calculated value). The calculated Bragg positions correspond to the short vertical lines (green), while the bottom trace represents the graph of the difference between the experimental and calculated profile. BCP = biphasic calcium phosphate; HAp = hydroxyapatite; β-TCP = β-tricalcium phosphate.

The structural parameters and mass fraction (wt%) of the crystalline HAp and β-TCP phases identified in the BCP were obtained by structural refinement using the Rietveld method and the computer program Fullprof [41]. For refinement, the diffraction profiles of HAp and B-TCP phases were indexed to Inorganic Crystal Structure Database (ICSD) standards: Ca_10_(PO_4_)_6_(OH)_2_ system hexagonal (space group P63/m) and Ca_3_(PO_4_)_2_ rhombohedral (space group R3c), respectively [41]. A good agreement is observed between the calculated (black solid line) and the experimental (red circles) profile, as can be seen in Figure 3. The bottom line (dashed) corresponds to the difference between the experimental values and those calculated by the theoretical model, while the vertical lines (green) show the Bragg positions calculated by the refinement. In addition, the GoF (Goodness of Fit), which is an indicator of the quality of fit, showed values of 1.4 and 1.8% for HAp and β-TCP phases, respectively. These values are lower than what is considered acceptable by the basic rule for this indicator, that is, GoF = (Rwp/Rexp) < 4% [42]. The lattice parameters, the wt% of HAp and β-TCP phases, and the quality factors of the fit are presented in Table 1. The wt% calculated by the Rietveld method shows that the bioceramic BCP is composed of 57.2% HAp and 42.8% β-TCP. The Ca/P molar ratio of the BCP powder, determined by Rietveld refinement, was 1.59.

### 3.3. Analysis of Scaffolds Porosity by SEM and EDS

**Figure 4:**
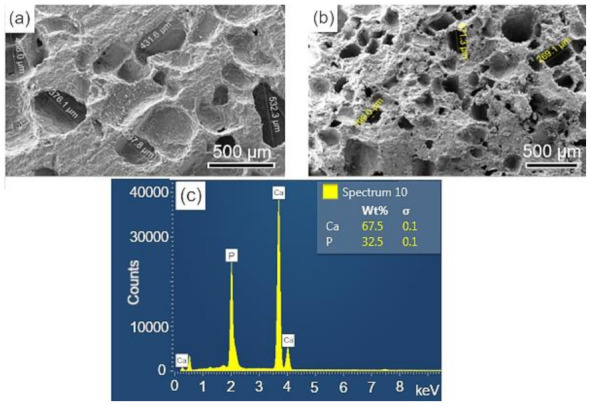
SEM of the scaffolds BCP sintered at 1100 °C for 8 h: (**a**) surface (sanded with 400 grit sandpaper); (**b**) fracture; (**c**) semi-quantitative elemental analysis by EDS performed on the scaffold surface resulted in a molar ratio (Ca/P) of 1.6. Ca = calcium; P = phosphorus; %wt = mass fraction; σ = mean deviation.

The surface and fracture micrographs (SEM) of the scaffolds are shown in Figure 4a,b. There are two types of pores in scaffolds: interconnected macropores, resulting from the elimination of spherical agglomerates of lactose, which have rounded morphology with diameters between 50 and 400 µm; and micropores in the pore walls, due to the BCP sintering stage. According to Hench et al. [48], for a biomaterial to be considered microporous, it should present interconnected pores with diameters between 50 and 250 μm because they favor cell and tissue penetration, as well as the development of a capillary network, essential for bone neoformation [48,49]. The open porosity of the scaffolds determined by the fluid displacement method (Archimedes Principle) was in the range of 57.21%. Figure 4c shows the results of the semi-quantitative chemical analysis by EDS performed on the scaffold surface (Figure 4a). The wt% of Ca (67.5) and P (32.5) resulted in a molar ratio of (Ca/P) of about 1.60, which is close to the value determined from the wt% of the HAp and β-TCP phases obtained via Rietveld refinement of 1.59.

### 3.4. BCP Powder Analysis by Infrared Spectroscopy (FTIR)

**Figure 5:**
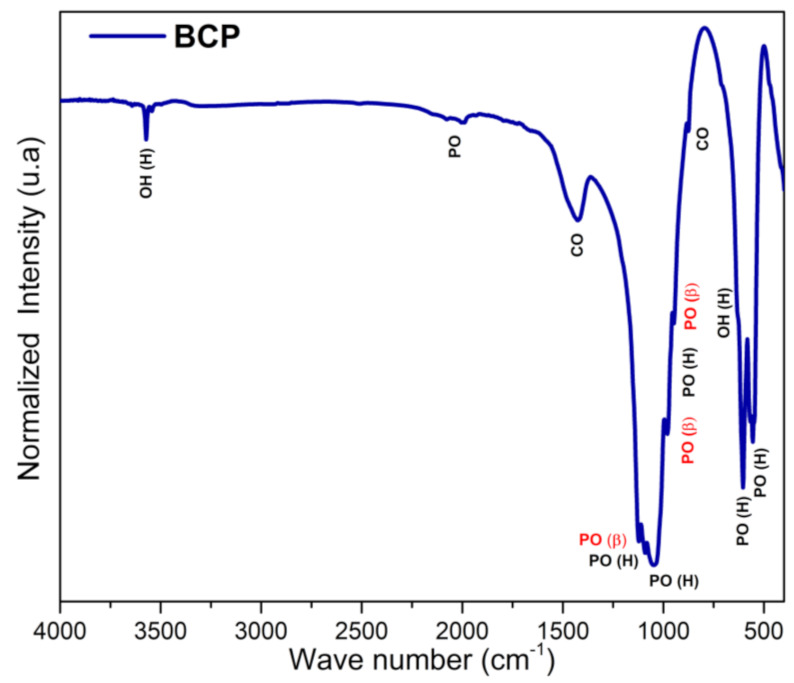
FTIR spectra of BCP powder obtained by calcination and milling of the fish bones. PO (PO_4_^3−^), OH (OH^−^), CO (CO_3_^2−^), H (Hap) and β (β-TCP). BCP = biphasic calcium phosphate.

The FTIR spectrum of BCP is shown in Figure 5 with observed vibrations associated with the functional groups PO_4_^3−^, CO_3_^2−^ and OH^−^. Bands associated with the non-degenerate symmetric stretching mode (ν_1_) of the PO_4_^3−^, group, which are related to β-TCP, are detected at 974 and 947 cm^−1^, while the one at 962 cm^−1^ is assigned to HAp. The bands associated with the asymmetric triple degenerate stretching mode (ν_3_) of the PO_4_^3−^ group and associated with the HAp phase are located at 1091, 1024 cm^−1^, while the one at 1120 cm^−1^ is assigned to the β-TCP phase. The triple degenerate strain mode (ν_4_) bands of the PO_4_^3−^ group, located at 631, 603 and 567 cm^−1^ are assigned to HAp, while the one at 551 cm^−1^ is associated with the β-TCP phase. The band at 470 cm^−1^ (weak) of the doubly degenerate strain mode (ν_2_) of the PO_4_^3−^ group is assigned to the HAp phase. The bands at 632 (weak) and 3572 cm^−1^, which are typical of HAp, are assigned to the libration (ν_L_) and stretching (ν_S_) modes of the OH^−^ group, respectively [50,51]. Bands of the antisymmetric stretching (ν_3_) and deformation (ν_2_) modes of the CO_3_^2−^ group are detected in the range between 1400 and 1600 cm^−1^ and at 875 cm^−1^, respectively [52].

### 3.5. BCP Powder Analysis by Micro-Raman

**Figure 6:**
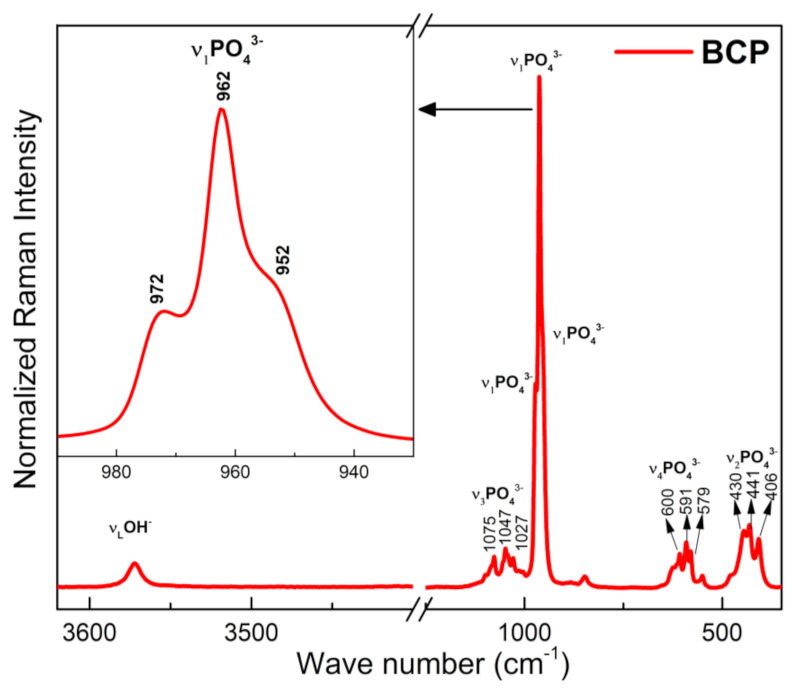
Raman spectra of the BCP powder obtained by calcination and milling of the fish bones. BCP = biphasic calcium phosphate.

The Raman spectrum of BCP is shown in Figure 6. Characteristic bands of the PO_4_^3−^ group are observed and related to the vibrational modes ν_1_, ν_2_, ν_3_ e ν_4_, of the HAp and β-TCP phases, with a strong overlap of the bands related to these two phases occurring in the ν_2_, ν_3_ e ν_4_ modes. The bands at 430, 441 and 406 cm^−1^ correspond to the doubly degenerate strain mode (ν_2_) of the (O-P-O) bond, while those located at 579, 591 and 600 cm^−1^ correspond to the triple degenerate strain mode (v_4_) of the (O-P-O) bond. The bands at 1027, 1047 and 1075 cm^−1^ are assigned to the antisymmetric triple degenerate stretching mode (ν_3_) of the (P-O) bond. The most intense bands in the spectrum, (highlighted), are assigned to the non-degenerate symmetric stretching mode (ν_1_) of the (P-O) bond. The band at 962 cm^−1^ is typical of the HAp phase, while those located at 952 and 972 cm^−1^ are assigned to the β-TCP phase. The band at 3572 cm^−1^, corresponds to the normal stretching mode (ν_S_), of the OH^−^ group and associated with the HAp phase [53,54].

### 3.6. Complexation of Curcumin with β-Cyclodextrin (β-CD)

The curcumin-β-CD complex by the co-precipitation method showed an efficiency value of 70% of the amount of curcumin that was initially added to the process. According to Mangolim et al. [35], the efficiency value of complexation by co-precipitation was 74% and, in addition, this process increased the solubility of the dye in water by 31 times, improved its stability to light by 18%, and was also 2.7 times more stable to pH variations.

### 3.7. In Vitro Study of the Bioactivities of Scaffolds

**Figure 7:**
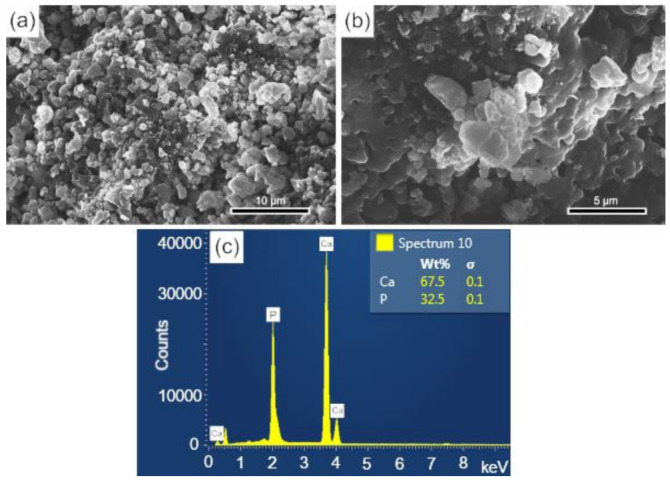
SEM images of the scaffold surface (control-BCP) sintered at 1100 °C for 2 h, before immersion in SBF: (**a**,**b**) morphology and microstructure of the surface and (**c**) EDS obtained on the sample surface. Ca = calcium; P = phosphorus; %wt = mass fraction; σ = mean deviation.

The surfaces of the scaffolds before and after immersion for 28 d in SBF were analyzed by SEM-EDS. In Figure 7a,b, at different magnifications, the surface morphology of the sintered scaffold is observed, whose microstructure corresponds to an intermediate stage of sintering with the neck formation and reduction of microporosity [36]. The EDS elemental analysis performed on the sample surface is shown in Figure 7c. The mass fraction (wt%) of calcium and phosphorus were used to calculate the Ca/P molar ratio of the BCP, which corresponded to a value of 1.60, a number close to that obtained by Rietveld refinement (1.59).

**Figure 8:**
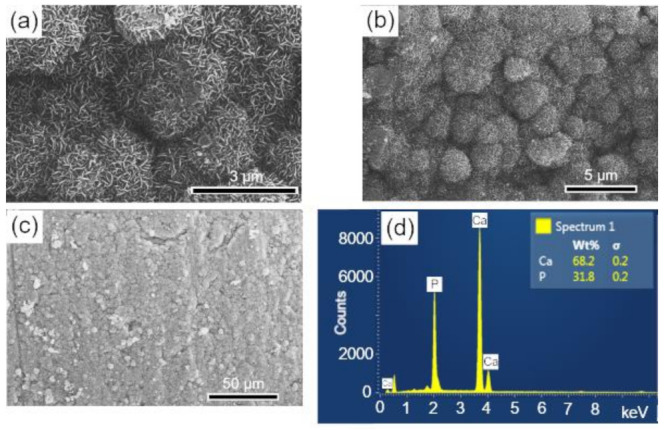
SEM micrographs of the scaffold surface (control-BCP) after 28 d of immersion in SBF, showing a characteristic morphology of apatite nucleation in bioactive materials: (**a**–**c**) images with different magnifications and (**d**) EDS obtained on the sample surface. Ca = calcium; P = phosphorus; %wt = mass fraction; σ = mean deviation.

**Figure 9:**
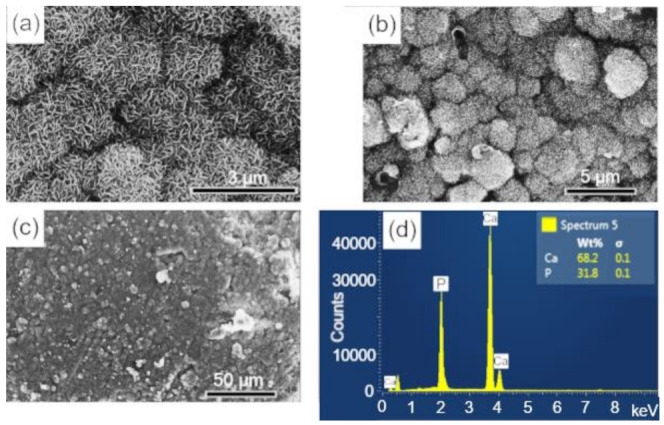
SEM imagens of the scaffold surface (BCP-CL) after 28 d of immersion in SBF, showing a characteristic morphology of apatite nucleation in bioactive materials: (**a**–**c**) images with different magnifications and (**d**) EDS obtained on the sample surface. Ca = calcium; P = phosphorus; %wt = mass fraction; σ = mean deviation.

**Figure 10:**
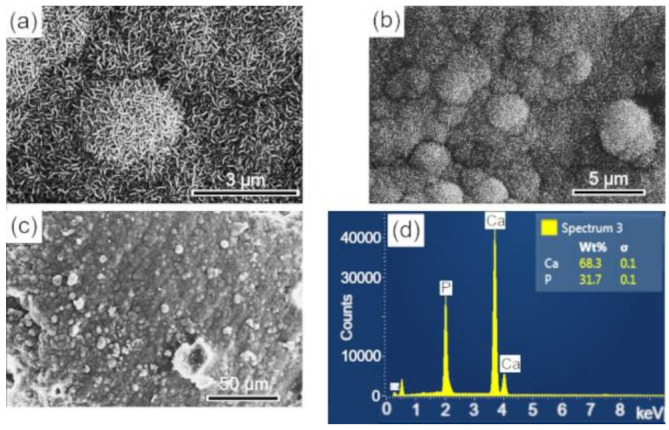
SEM imagens of the scaffold surface (BCP-CD.) after 28 d of immersion in SBF, showing a characteristic morphology of apatite nucleation in bioactive materials: (**a**–**c**) images with different magnifications and (**d**) EDS obtained on the sample surface. Ca = calcium; P = phosphorus; %wt = mass fraction; σ = mean deviation.

The surfaces of the scaffolds after immersion in SBF were analyzed by SEM-EDS, to evaluate the apatite formation (CaPs) on the surface of these samples. The results are presented in the sequence: BCP (Figure 8); BCP-CL (Figure 9) and BCP-CD (Figure 10). After 28 d of immersion, without fluid renewal, the formation of an apatite layer was observed on the surface of all the samples analyzed, whose morphology can be seen in the corresponding micrographs. At higher magnifications, a morphology composed of apatite nuclei consisting of short micro-stems was observed, as well as clusters of these nuclei, which are characteristic of apatite nucleation and growth in SBFs solution [44].

The EDS analysis showed that the molar ratio (Ca/P) of the material precipitated on the surface of the samples corresponded to values of 1.657, 1.657 and 1.662, respectively, which are very close to the HAp molar ratio (1.667) and higher than that observed in the BCP sample before immersion of 1.60. These results may indicate the dissolution of the β-TCP phase during the immersion process in SBF for 28 d, that is, its decomposition to form the apatite layer on the sample surface [37]. According to Zhang et al. [55], surface bioactivity is related to calcium phosphate precipitation and mineralization, which are affected by the physical and chemical properties of the surface. The processes of nucleation and growth of apatite on the sample occur with the dissolution of its surface and precipitation of the apatite layer [55].

In summary, we observed that after 28 d of immersion in SBF, there was nucleation (precipitation) of an apatite layer on the surface of the three samples analyzed, with similar characteristics and showing the bioactivity of the materials analyzed.

### 3.8. Ex Vivo Micro-Raman Analysis of Implants

In order to investigate the optical absorption of the β-TCP phase as a function of the implantation time, the micro-Raman analysis was directed to the spectrum region between 1000 to 900 cm^−1^ because the β-TCP and HAp phases are most evident in this spectral range. The absorption spectrum of β-TCP can be seen in Figure 6.

**Figure 11:**
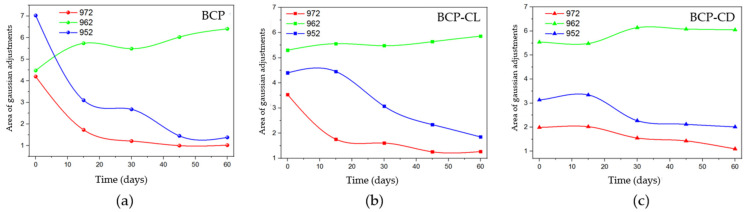
Behavior of the areas of the adjusted bands in the Micro-Raman analysis as a function of the analyzed periods, being the band 962 cm^−1^ referring to HAp and the bands 972 cm^−1^ and 952 cm^−1^ referring to β-TCP. (**a**) BCP, shows the most evident decay in the first 15 d of study of the bands related to β-TCP; (**b**) BCP-CL and (**c**) BCP-CD show that the bands related to β-TCP did not present evident decay in the first 15 d, but slow throughout the 60 d of study.

The behavior for the bands referring to the β-TCP phase (Figure 11) was obtained after performing Gaussian fits for the BCP, BCP-CL and BCP-CD groups. The bands at 972 and 952 cm^−1^ decreased over time. When the groups are compared with each other, the BCP group (Figure 11a) has a pronounced decrease until 30 d after implantation, with the greatest reduction in the first 15 d. Figure 11b,c shows that in the BCP-CL and BCP-CD groups, the decay of the β-TCP bands were less pronounced over time. Comparing the bands of the scaffolds impregnated with free and complexed curcumin with β-CD. It can be seen that the decay of β-TCP in those with free curcumin was slightly more pronounced after 30 d them complexed curcumin with β-CD, suggesting that the controlled release of curcumin interfered with the decay of the bands of β-TCP.

As observed in the behavior of the bands as a function of the analyzed implantation times, it can be suggested that, in the scaffolds impregnated with free or complexed curcumin, there may have been a decrease in the dissolution of the β-TCP phase. The local increase in the ionic concentration produced by the ceramic solubility has a positive impact on the proliferation and differentiation of osteoblasts, as well as on the bone formation process [31,56]. However, both curcumin and β-CD can bind to these ions, interfering with bone formation. Bose et al. [56] suggested that curcumin has chelating ability, and can bind to the calcium ions of HAp-based scaffolds, reducing their solubility and affecting osteogenesis, which is induced by Ca^2+^ and PO_4_^3−^ ions present in body fluids. Liang et al. [57] demonstrated that the surfaces of β-TCP crystals are positively charged, generally due to the presence of Ca^2+^ ions near the surfaces [57]. On the other hand, the outer surface of the β-CD molecules is hydrophilic, so that the OH- groups in this region can bind to the Ca^2+^ ions of the calcium phosphate ceramics, reducing the ionic bioavailability, and its effect on the bone regeneration [58,59]. Based on these authors, the results of this study suggest that β-TCP had a slower ionic release as a result of interactions with other molecules in the system.

### 3.9. Histological Study of the Implants

Osteogenesis occurred in the scaffolds of the three groups studied. There was no difference in morphology or amount of bone tissue in the scaffolds, comparing the three groups. First, the pores have been colonized by vascularized connective tissue from the surrounding periosteum and dura mater. Osteogenesis occurred predominantly by intramembranous ossification, typical of the calvaria, but the presence of hyaline cartilage in some pores showed also endochondral ossification.

**Figure 12:**
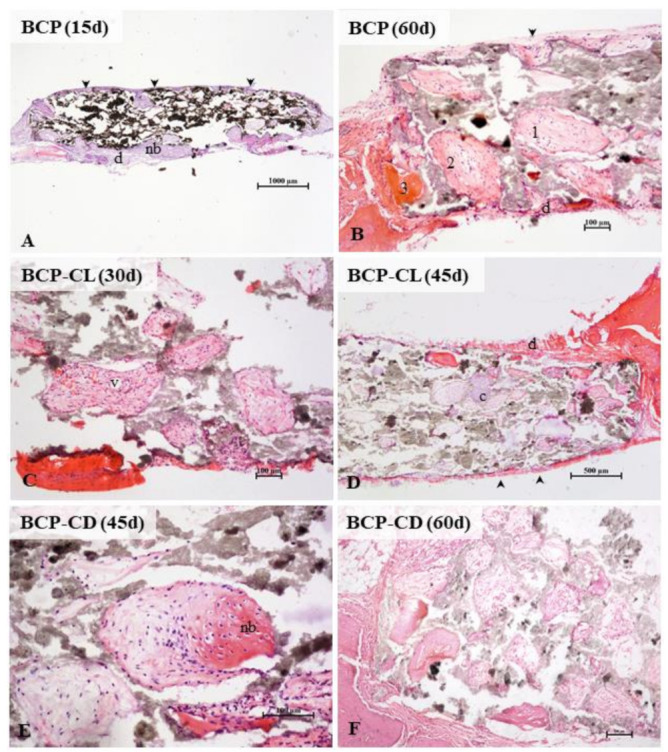
Photomicrographs of critical size defects in rat calvaria after implants with scaffolds of BCP (**A**,**B**), BCP-CL (**C**,**D**) and BCP-CD (**E**,**F**). The animals remained with the implants for 15 (**A**), 30 (**C**), 45 (**D**,**E**) or 60 (**B**,**F**) d. The scaffold pores of the three groups studied were filled by cells and blood vessels (v) from the periosteum (arrowhead) and dura mater (d). Intramembranous ossification, typical of the calvaria, was the main type. Initially, a loose connective tissue filled the pores. A primary bone, with denser fibers, irregularly disposed, was deposited from the margins of the pores, followed by lamellar deposition of bone matrix. In the three groups studied, the presence of pores filled with hyaline cartilage was occasionally observed (c). In (**A**), a panoramic view of a BCP scaffold shows most of the still empty pores and filled peripheral pores. In (**B**) observe the detail of collagen deposition in the pores. The numbers 1, 2, and 3 (**B**,**C**) indicate the gradation in the ossification process. The pore (1) shows the beginning of densification of the loose connective tissue, in (2) larger collagen fiber deposition in the periphery of the pore (primary bone tissue), and in (3), the pore filled with mature lamellar bone. At 45 (**D**,**E**) and 60 (**B**,**F**) d, more central pores presented colonized by connective tissue, but with little lamellar bone formation. In (**E**), the detail of a pore in the process of ossification. BCP = biphasic calcium phosphate; CL = free curcumin; CD = curcumin complexed with cyclodextrin; d = dura mater; nb = new bone; arrow head = periosteum; v = blood vessel; c = cartilage. Staining: hematoxylin and eosin.

In the three groups analyzed, the microscopic analysis (Figure 12) showed, over time, a greater frequency of pores filled with bone tissue. At 60 d of study, all pores had some content, being mostly connective tissue in different degrees of maturation, and only a small amount of pores, especially the marginal ones, were totally or partially occupied by mature bone tissue. There was no complete regeneration of any defect in 60 d. Studies have shown that the implantation of porous or non-porous biocomposites in critical-size defects in the calvaria of rats stimulated bone formation, but without promoting complete regeneration even after 90 d of observation [20,21,60,61].

According to the literature, curcumin is not an inert molecule for bone tissue. In in vivo studies, it has been demonstrated potential biological activity on bone tissue.

When administered intraperitoneally, curcumin stimulated bone formation in the rat model of femoral fractures [62]. After oral use, it had positive effects on osteoporosis, attenuating bone lesions [38] and osteopenia, with improvement in bone mineral density [32]. The implantation of curcumin-impregnated scaffolds in a rat distal femur fracture model stimulates bone matrix mineralization, in addition to improving osteogenic and angiogenic capacity in bone regeneration [56].

Although the role of curcumin in bone metabolism is controversial, studies have shown that the use of curcumin, similar to BMP-2 and growth factors, can induce osteoblastic differentiation [31,63,64] and increase the expression of osteocalcin, a marker of bone formation, and has a regulatory role in bone resorption [31,38]. In contrast, Notoya et al. [65] demonstrated that curcumin inhibits the proliferation of osteoblastic cells from the calvaria of rats, inducing the death of human cancerous osteoblasts.

In this study, we expected a better bio performance of the scaffolds with curcumin, considering the already known osteogenic potential of BCP, especially by the β-TCP phase, in addition to the described biological activity of curcumin on bone tissue. We believed that the slow release of curcumin complexed with β-CD could potentiate, in a controlled manner, the osteogenic stimulus in the scaffolds. The micro-Raman results showed that β-TCP dissolution was slower in curcumin-impregnated scaffolds, however, this was not reflected in vivo osteogenesis. Based on the literature [56,57], we can infer that after its dissolution, the ions of the β-TCP molecule were captured by curcumin itself, compromising its availability for bone formation.

## 4. Conclusions

Bones from the Nile tilapia are an excellent source for obtaining and producing natural bioceramics. In this study, the BCP used presented the physical–chemical and structural characteristics for a good biomaterial. The presence of free curcumin and complexed with β-CD in BCP demonstrated the ability to induce bone-like apatite crystallization in vitro, with results similar to BCP control. In vivo, qualitative analysis showed that the incorporation of curcumin did not provide additional effect to the studied bioceramics. However, the decrease in the release of β-TCP phase with the presence of curcumin, and especially the one complexed with β-CD, may be an interesting feature for bone tissue engineering, and could also be an alternative for the development of a prolonged release system of β-TCP phase.

## Figures and Tables

**Table 1:** Fractional mass (wt%), quality factors and structural parameters obtained by the Rietveld refinement.

Phase: β-TCP—(Ca_3_(PO_4_)_2_) System: Rhombohedral—R3*c* (167)					Phase: HAp—(Ca_10_(PO_4_)_6_(OH)_2_) System: Hexagonal—*P*63/*m* (176)				
a = b (Å)	c (Å)	V (Å^3^)	ρ g/cm^3^	wt%	a = b (Å)	c (Å)	V (Å^3^)	ρ g/cm^3^	wt%
10.3480(6)	37.071 (2)	3457.9 (4)	3.09	42.8	9.422(4)	6.881 (3)	529.30(4)	3.142	57.2
10. 4290 *	37.380 *	3520.91 *	3.0 *	100*	9.418 **	6.884 **	528.0 **	3.16 **	100 **

* jcpds 09-0169 ** jcpds 09-0432.

## Data Availability

Data available upon request.

## Sample Availability

Samples of the compounds are not available from the authors.
